# Effects of Supplementation with Encapsulated Different Postbiotics, Alone or with Inulin, on Growth Performance, Carcass and Organ Characteristics, Blood Parameters, Growth Hormone, and Insulin-like Growth Factor mRNA in Broilers

**DOI:** 10.3390/ani15071010

**Published:** 2025-03-31

**Authors:** Helin Atan Çırpıcı, Figen Kırkpınar

**Affiliations:** Department of Animal Science, Faculty of Agriculture, Ege University, İzmir 35100, Türkiye; figen.kirkpinar@ege.edu.tr

**Keywords:** broiler, carcass traits, encapsulated, growth performance, inulin, postbiotic

## Abstract

This study aimed to evaluate the effects of encapsulated postbiotics derived from various probiotic microorganisms, alone or in combination with inulin, on the growth performance, carcass traits, organ weights, blood parameters, and mRNA expression of selected hormones in broilers. A total of 588 one-day-old male Ross-308 chicks were randomly designated to six replicates of seven dietary treatments (initial body weight: 40.85 ± 0.56 g, per replicate, n = 14 chicks). The treatments consisted of a basal diet (C), supplemented with encapsulated postbiotics (0.30%) derived from *Lactobacillus plantarum* (ELP), *Bacillus subtilis* (EBS), or *Enterococcus faecium* (EEF), as well as combinations of these encapsulated postbiotics with 1.0% inulin (ELPI, EBSI, and EEFI) for six weeks. The results demonstrated that the body weight and body weight gain of birds that were fed diets supplemented with encapsulated postbiotics or their combinations with inulin significantly increased in comparison to the C group (*p* < 0.001). Feed intake (FI) remained unaffected during days 1–21, as did feed conversion ratios (FCR) during days 22–42, and days 1–42 demonstrated no significant differences (*p* > 0.05). However, FCR improved during days 1–21, and FI increased during days 22–42 and 1–42 (*p* < 0.05). Carcass yield, including breast, thigh, and abdominal fat yields, was enhanced (*p* < 0.001). Although the relative weights of the heart, spleen, pancreas, and liver were unaffected (*p* > 0.05), the relative weight of the bursa of Fabricius increased (*p* < 0.001). Serum antioxidant status and immunoglobulin A and M levels were higher, while liver enzymes, cholesterol, triglycerides, and total oxidant status were lower in the supplemented groups compared to the control group (*p* < 0.001). Serum glucose and protein levels remained unchanged (*p* > 0.05). The mRNA expression of growth hormone and insulin-like growth factor was upregulated in the supplemented groups (*p* < 0.001). In conclusion, encapsulated postbiotics (0.30%) derived from different probiotics, alone or combined with inulin (1.0%), positively influenced growth performance, carcass traits, and immunity in male broilers.

## Simple Summary

The poultry industry is one of the most dynamic sectors of agriculture, and the broiler industry has contributed to providing the growing global demand for animal protein. The use of antibiotic growth promoters (AGPs) as growth performance and feed conversion ratio enhancers has long been an accepted practice. However, due to concerns regarding antibiotic resistance and consumer health, AGPs have been banned in many countries, compelling the search for effective alternatives. Postbiotics, metabolite compounds derived from probiotics, have gained attention as promising feed additives due to their potential benefits in improving gut health, immunity, and growth performance. This study investigated the effects of encapsulated postbiotics (0.30%) derived from different probiotics, alone or in combination with inulin (1.0%), on male broilers. The results demonstrated that encapsulated postbiotics (0.30%) derived from different probiotics, either alone or in combination with inulin (1.0%), significantly improved growth performance, carcass characteristics, and immune responses in male broilers.

## 1. Introduction

It has been reported that the human preference for chicken meat as a source of protein has increased by 70% over the past 30 years [1]. This rising demand highlights the need for environmentally friendly and sustainable animal products, which requires a more efficient use of natural resources while meeting consumers’ expectations of safe food. Within the framework (BAT) of Directive 2010/75/EU of the European Parliament and the European Council [2], the adoption of certain feeding strategies, such as feed additives, has been mandated to enhance production performance. Antibiotics have been widely used as growth promoters in the broiler industry for many years [3]. However, the extensive and uncontrolled use of antibiotics has led to the development of antibiotic resistance in pathogens and increased the risk of antibiotic residues in animal products, raising concerns among consumers [4]. Because of these adverse effects, the use of antibiotics as growth promoters in the livestock sector has been banned in many countries. This ban has intensified the search for alternative feed additives.

Biotics are feed additives that exert regulatory, protective, and activating effects on microbiota. They can be classified into four categories: prebiotics, probiotics, postbiotics, and synbiotics [5]. Probiotics have a long history of use as feed additives in broiler diets. These additives are a popular approach to improving animal production performance, immune system function, microbiota balance, and overall health and welfare in animals [6]. Many species and strains of probiotics with varying mechanisms of action are now available on the market.

*Lactobacillus plantarum* is a highly valued probiotic due to its ability to produce antifungal metabolites, including proteinaceous compounds, hydroxyl fatty acids, and phenolic compounds. It also exhibits antioxidant, anti-cancer, anti-proliferative, anti-obesity, anti-inflammatory, and anti-diabetic properties [7]. Supplementing broiler diets with *Lactobacillus plantarum* has been reported to regulate intestinal microbiota, enhance disease resistance, nutrient digestion and absorption, and the proliferation of beneficial bacteria, and reduce the prevalence of pathogenic bacteria [8,9].

*Bacillus subtilis* is another probiotic widely utilized due to its antimicrobial, antioxidant, immunomodulatory, antibacterial, anti-inflammatory, and antifungal properties [10,11]. It also exhibits high enzyme production during fermentation. Supplementing broiler diets with *Bacillus subtilis* strengthens the immune system, enhances nutrient digestibility, and improves intestinal morphology, microbiota composition, and overall functionality [12,13].

*Enterococcus faecium* is another group of probiotics, belonging to the endogenous intestinal microbiota of both humans and animals [14]. Studies have demonstrated that supplementing mixed poultry diets with *Enterococcus faecium* stimulates the growth of lactic acid bacteria (LAB) in the small intestine, improves intestinal morphology, and enhances FCR. It can also beneficially modulate the cecal microbiota [15,16]. Additionally, *Enterococcus faecium* can adhere to the intestinal tract of animals, inhibiting pathogenic bacteria such as *Salmonella*, *Staphylococcus aureus*, and *Escherichia coli* (*E. coli*) [17]).

Postbiotics are metabolite compounds and/or products synthesized during the growth, reproduction, or fermentation of probiotic microorganisms. These metabolite-derived compounds and/or products are considered to have mechanisms of action similar to those of probiotics [18]. Postbiotics include bacteriocins, short-chain fatty acids (SCFAs), proteins, peptides, and enzymes, depending on the probiotic strain and species from which they are derived [19]. Previously conducted studies have reported that the use of postbiotics produced from *Bacillus subtilis* [20,21] and *Lactobacillus plantarum* [5,22,23] as feed additives in broiler diets has inhibitory effects on pathogenic bacteria (*E. coli*, *Salmonella typhimurium*, and *Listeria monocytogenes*) while improving growth performance, immune function, animal health, and meat quality. Humam et al. [18] observed that the addition of postbiotics derived from different *Lactobacillus plantarum* strains to the finisher diet of broiler chickens under heat stress improved growth performance, intestinal morphology, microbiota, and SCFA levels. Among the strains tested, postbiotics derived from strain RI11 demonstrated superior effects compared to those derived from RG14 and UL4. The researchers, thus, concluded that the composition and mechanisms of action of postbiotics can vary depending on the strain. However, no studies comparing postbiotics derived from different probiotic species have been found in the literature.

Prebiotics are non-digestible feed components that stimulate the activity of beneficial gut bacteria (*Bifidobacteria* and *Lactobacilli*) and growth while inhibiting the proliferation of harmful pathogens (*E. coli* and *Salmonella*) [24]. A typical example of a prebiotic is inulin. Inulin also enhances nutrient (especially mineral) absorption by promoting the growth of beneficial bacteria, lowering the environmental pH (through increased SCFA production), and increasing the surface area of intestinal epithelial cells. Kareem et al. [24] reported that the use of postbiotics in combination with inulin improved growth performance, animal health, and microbiota composition in broilers compared to the use of postbiotics alone. This mechanism of action is attributed to the role of inulin in supporting gut health and the bioactive metabolites present in postbiotics, such as bacteriocins and SCFAs. Inulin exerts beneficial luminal and systemic effects on the host as a result of fermentation, including enhancing microbiota balance, forming a protective barrier against intestinal pathogens, stimulating the immune system, and improving production performance [25,26]. Metabolite by-products of postbiotics, particularly SCFAs, exhibit anti-inflammatory properties and activate immune cells such as T cells and macrophages [5]. The combination of inulin-induced immune stimulation and the anti-inflammatory effects of postbiotics may enhance immune responses and reduce the risk of infection.

Microencapsulation is a technology employed to preserve the quality of sensitive substances, such as enzymes and amino acids, while enabling the development of materials with new and valuable properties [27]. This method is increasingly applied to feed additives to enhance product profitability and shelf life, ensure the controlled release of active compounds, and protect these compounds from environmental disturbances. Natsir et al. [28] found that encapsulating probiotics ensures their homogeneous distribution in mixed feed, enhances their stability during storage and processing, and facilitates their passage through the gizzard and intestines without degradation. Moreover, some researchers have reported the beneficial effects of using encapsulated SCFAs [29], encapsulated organic and encapsulated essential oils [30], and encapsulated enzymes [31] in broiler chickens. In this study, we preferred to use the encapsulated form of postbiotics due to their content of SCFAs, enzymes, and other metabolites.

Against this background, encapsulated postbiotics derived from different probiotic microorganisms, either alone or in combination with inulin, show promise as feed additives that confer probiotic effects, serve as alternatives to antibiotics, and enhance feed efficiency. This study evaluated the potential of encapsulated postbiotics for cost-effective, welfare-friendly, and environmentally sustainable broiler production by improving growth performance, immune function, and intestinal health. Specifically, the study aimed to investigate the effects of adding encapsulated postbiotics derived from *Lactobacillus plantarum*, *Bacillus subtilis*, and *Enterococcus faecium* (0.30%), either alone or in combination with inulin (1.0%), to the basal mixed diet of male broilers throughout 42 days.

## 2. Materials and Methods

### 2.1. Animal Ethics

The trial protocol and all procedures were conducted in strict accordance with ethical standards and approved by the Farm Animal Experiments Local Ethics Committee of the Faculty of Agriculture, Ege University (Approval No: 2022/001). The committee ensures that the use and care of research animals are both ethical and humane.

### 2.2. Preparation of Postbiotics Derived from Probiotics Strain and Encapsulation Process

The stock cultures of *Lactobacillus plantarum* (DSM 1055), *Bacillus subtilis* (DSM 5611), and *Enterococcus faecium* (NCIMB 10415) were prepared at the Laboratory of Hektaş High Technology Center (Ankara, Turkey). *Lactobacillus plantarum* and *Enterococcus faecium* were cultured on de Man Rogosa Sharpe agar (MRS; Biolife, Milano, Italy), while *Bacillus subtilis* was cultured on Nutrient Agar (Lansing, MI, USA). They were incubated at 30 °C for 48 and 24 h, respectively. The stock cultures of probiotics were prepared as described by Kareem et al. [24]. For the fermentation strains, an inoculum size of approximately 10^9^ colony-forming units per milliliter (CFU/mL) was achieved at the specified optical density (OD600), and the cultures were incubated for 24 h. After incubation, the cultures were centrifuged (10,000 rpm at 4 °C for 15 min). Postbiotics were collected using 0.22 mm filter paper (Sartorius Minitart, Gottingen, Germany). Postbiotics were encapsulated using ionic gelation/extrusion to determine the optimal concentrations of sodium alginate (1.5, 2, 2.5, 3, 3.5, and 4% *w*/*v*) and calcium chloride (CaCl_2_: 0.25, 0.5, 1.0, 2.0, and 4 M). The encapsulation process was conducted based on the modified methods of Reineccius [32] and Gouin [33]. Briefly, postbiotics were encapsulated at room temperature using 0.5 M CaCl_2_ and 2% *w*/*v* sodium alginate. The encapsulated postbiotics were filtered and left to dry naturally for 1 h. Encapsulated postbiotics were stored at 4 °C until they were added to the basal diet. Inulin (Sigma-Aldrich, St. Louis, MO, USA, No. I2255) was obtained from the commercial market and used as a feed additive.

### 2.3. Animals, Housing and Experimental Design

In this study, a total of 588 one-day-old male Ross 308 broiler chicks, with an initial body weight of 40.85 ± 0.56 g, were randomly distributed into seven treatment groups. Each group consisted of six replicates, with 14 birds per replicate. The treatment groups were as follows: a basal diet (C), a basal diet supplemented with 0.30% encapsulated postbiotics derived from *Lactobacillus plantarum* (ELP), a basal diet supplemented with 0.30% encapsulated postbiotics derived from *Bacillus subtilis* (EBS), a basal diet supplemented with 0.30% encapsulated postbiotics derived from *Enterococcus faecium* (EEF), a basal diet supplemented with 0.30% encapsulated postbiotics derived from *Lactobacillus plantarum* and 1.0% inulin (ELPI), a basal diet supplemented with 0.30% encapsulated postbiotics derived from *Bacillus subtilis* and 1.0% inulin (EBSI), and a basal diet supplemented with 0.30% encapsulated postbiotics derived from *Enterococcus faecium* and 1.0% inulin (EEFI). Based on the findings of a study conducted by Kareem et al. [24] the inulin supplementation level was set at 1.0%. Feed and water were provided ad libitum until the birds reached 42 days of age. Starter and finisher diets (Table 1) were offered from days 1 to 21 and days 22 to 42, respectively. The basal feeds were in granulated form, and the feed additives were incorporated into the basal feeds using a pre-mixer. The environmental temperature was initially set at 32 ± 1 °C and gradually reduced until it reached 24 ± 1 °C. The lighting program consisted of 23 h of light and 1 h of darkness (for 42 days), while the relative humidity was kept at 65%. Each 1.4 m × 1.2 m floor pen was furnished with wood shavings as bedding material, one round feeder, and four to five nipple drinkers to ensure adequate access to feed and water.

### 2.4. Growth Performance Data Collection

The body weight (BW) and feed intake (FI) of the male broilers were evaluated on a replicate cage basis on days 1, 21, and 42 of the study. These measurements were employed to calculate body weight gain (BWG) over three distinct periods: days 1 to 21, days 22 to 42, and days 1 to 42. Additionally, feed efficiency was assessed by determining the FCR based on the FI and BWG data.

### 2.5. Carcass Characteristics and Internal Organs

On day 42, 12 animals were selected from each treatment group, closely matching the average body weight (two birds/replicate). After 8 h of feed and water withdrawal, the broilers were humanely euthanized by severing the jugular vein. The birds were scalded at 58 °C, de-feathered, eviscerated, and then chilled. The body parts, including the carcass, breast, thighs, abdominal fat, and internal organs (heart, liver, spleen, bursa of Fabricius, and pancreas), were dissected and weighed individually. All carcass parts and internal organs were expressed as percentages of the empty body weight using the following formula:

Cut yield (%) = (Weight of cut/Empty body weight) × 100

### 2.6. Collection of Blood Samples for Serum Biochemical Analyses

At the time of slaughter, approximately 10 mL of blood was collected from the vena jugularis and transferred into heparin tubes (Greiner Bio-One, Les Ulis, France, [34]). The blood samples were then centrifuged (4000 rpm at 10 °C for 10 min). Serum concentrations of aspartate aminotransferase (AST; Otto Scientific, kit no: OttoBC127, Ankara, Turkey), alanine aminotransferase (ALT; Otto Scientific, kit no: OttoBC128, Ankara, Turkey), alkaline phosphatase (ALP; kit no: OttoBC124, Ankara, Turkey), gamma-glutamyl transferase (GGT; Otto Scientific, kit no: OttoBC141, Ankara, Turkey), lactate dehydrogenase (LDH; Otto Scientific, kit no: OttoBC129, Ankara, Turkey), cholesterol (Chol; Otto Scientific, kit no: OttoBC135, Ankara, Turkey), triglycerides (TG; Otto Scientific, kit no: OttoBC155, Ankara, Turkey), glucose (Glu; Otto Scientific, kit no: OttoBC142, Ankara, Turkey), total protein (TP; Otto Scientific, kit no: OttoBC154, Ankara, Turkey), total oxidant status (TOS; REL ASSAY, kit no: RL0024, Gaziantep, Turkey), total antioxidant status (TAS; REL ASSAY, kit no: RL0017, Gaziantep, Turkey), immunoglobulin A (IgA; Otto Scientific, kit no: OttoBC146, Ankara, Turkey), and immunoglobulin M (IgM; Otto Scientific, kit no: OttoBC149, Ankara, Turkey) were determined using the MINDRAY-BS400 (Maharashtra, India) via the colorimetric method.

### 2.7. mRNA Extraction of GHR and IGF-1 Genes

Immediately after slaughter, liver samples were rapidly excised, weighed, flash-frozen (30 mg) in liquid nitrogen, and stored at −80 °C until the conduction of the analysis. In sum, TriPure solutions were added to each liver sample, which was then mechanically homogenized. Following homogenization, 200 µL of chloroform was added to the samples, and they were incubated at room temperature for 5 min. The samples were then centrifuged at 12,000 rpm at 4 °C for 20 min. The supernatants were transferred into new transparent-top tubes, and isopropanol was then added. After incubating the samples at room temperature for 10 min, they were centrifuged at 12,000 rpm at 4 °C for 10 min. The supernatants were discarded, and 75% ethanol was added to each plate. Following a final centrifugation at 7500 rpm at 4 °C for 5 min, the excess ethanol was evaporated, and the samples were resuspended in RNA-nuclease-free water. The purity and concentrations of the RNA samples were determined using a Nanodrop 2000c spectrophotometer (Thermo Scientific, Wilmington, DE, USA). The absorbance ratio (A260/A280) for all RNA samples ranged between 1.8 and 2.0. cDNA was produced from the RNA samples using the RevertAid RT Reverse Transcription Kit (Thermo Scientific, Wilmington, DE, USA). Real-time PCR analysis was conducted on the Roche LightCycler 480 II using the LightCycler^®^ 480 SYBR Green I Master (Roche Applied Science, Mannheim, Germany). Primer sequences for the target genes IGF-1 and GHR, and the housekeeping gene ACTB, were specifically designed using the NCBI and ENSEMBLE gene databases (Table 2). Primer specificity was confirmed using the BLAST program (version 2.13.0). Data obtained from real-time PCR were analyzed using both absolute quantification and advanced relative quantification methods. For relative quantification, Ct values of the target genes were normalized to ACTB, and normalized values were compared with those of the control groups. Fold change values were calculated, with significant downregulation defined as <0.5 and significant upregulation as >2.

### 2.8. Statistical Analyses

Sample size estimation was conducted using GPower 3.1.9.7. The analysis indicated that a minimum of 84 animals per group (total: 588 animals) was required to achieve a study power exceeding 80% at a 95% confidence interval, with a significance level of 0.05 and a medium effect size.

For all data, the pen was considered to be the experimental unit. The normality of the data was assessed through the Kolmogorov–Smirnov test, while the homogeneity of variances was determined with Levene’s test. The data were analyzed using one-way ANOVA with IBM SPSS Statistics version 25 [35]. Significant differences among means were determined using Duncan’s multiple range test, with significance accepted at *p* ≤ 0.05. Duncan’s multiple range test was chosen for post hoc comparisons due to its sensitivity in detecting differences among groups while controlling Type I errors, particularly in analyses involving a large number of groups.

## 3. Results

The effect of dietary supplementation of encapsulated postbiotics derived from different microorganism species alone or with inulin on broiler growth performance is presented in Table 3. No mortalities among the birds occurred during the experimental period. Compared to the C group, groups with encapsulated postbiotics alone or with inulin additives increased (*p* < 0.001) in BW on 21 and 42 days and in BWG between 1 and 21 days. These values were at their highest (*p* < 0.001) in the EBSI and EBS groups, followed by the ELPI, ELP, EEFI, and EEF groups, respectively. At 42 days, no statistically significant difference was observed in BW among the ELP, EEF, ELPI, and EEFI groups. Birds that were fed encapsulated postbiotics alone or with inulin had higher (*p* < 0.05) BWG and FI between days 22–42 and 1–42 compared to those fed control diets. BWG between days 1 and 42 was highest in the EBS and EBSI groups, significantly lower in the ELP, EEF, ELPI, and EEFI groups, and lowest in the C group. There was no significant difference (*p* > 0.05) in FI among the treatments from days 1 to 21. The best FCR during the 1–21 day-period was observed in the EBSI, EBS, and ELPI groups, while the worst FCR was found in the C group (*p* < 0.001). However, there were no statistically significant differences between the ELPI group and the EEFI, EEF, and ELP groups. However, FCR between days 22–42 and 1–42 did not differ (*p* > 0.05) across all groups.

The effect of the dietary supplementation of encapsulated postbiotics derived from different microorganism species alone or with inulin on broiler carcass characteristics is depicted in Table 4. Birds fed with the addition of encapsulated postbiotics alone or with inulin had higher (*p* < 0.001) carcass, breast, and thigh weight (g) and yield (%) compared to the C birds. The highest of these values were recorded in the (*p* < 0.001) EBSI and EBS groups, followed by the ELPI, ELP, EEFI, and EEF groups. The highest (*p* < 0.001) thigh yield was observed in the EBSI and EBS groups, followed by the EBS, EBSI, ELP, and ELPI groups, with no statistically significant differences observed among them. The supplementation of postbiotics in encapsulated form alone or with inulin did not affect (*p* > 0.05) abdominal fat weight, whereas it reduced (*p* < 0.001) abdominal fat yield (%) compared to the C group.

The influence of the dietary supplementation of encapsulated postbiotics derived from different microorganism species alone or together with inulin on relative weight (BW/organ weight, %) of the heart, liver, spleen, bursa of Fabricius, and pancreas is exhibited in Table 5. Supplementation of encapsulated postbiotic or with inulin in the diets increased (*p* < 0.05) the relative weight of bursa of Fabricius. However, there was no significant (*p* > 0.05) difference among the treatments in the relative weights of the heart, spleen, liver, and pancreas.

The effect of dietary supplementation of encapsulated postbiotics derived from different microorganism species alone or with inulin on some components in blood serum (AST, ALT, ALP, GGT LDH, Chol, TG, Glu, TP, TAS, TOS, IgA, and IgM) is depicted in Table 6. Statistically significant differences were observed (*p* < 0.001) regarding the AST, ALT, ALP, GGT, and LDH levels of the groups. In general, no significant effect of inulin supplementation was detected in the experimental groups (*p* > 0.05). All experimental groups were found to have significantly lower AST values in blood serum than the control (*p* < 0.001). The lowest (*p* < 0.001) AST values in blood serum were recorded in the EBS, EBSI, ELP, and ELPI groups, followed by the EEF and EEFI groups. The ALT and ALP values of the experimental groups supplemented with feed additives were similar among the EBSI, EBS, ELPI, ELP, EEFI, and EEF groups. They were lower compared to the C group (*p* < 0.001). The highest (*p* < 0.001) GGT value was recorded in the C group, significantly lower and similar values were observed among the EEF, EEFI, ELP, ELPI, and EBS groups, and the lowest (*p* < 0.001) values were observed in the EBSI group. The highest (*p* < 0.001) LDH value was recorded in the C group, followed by the EEF and EEFI groups, with the EEFI group having values similar to those of the ELP group. The lowest (*p* < 0.001) LDH values in serum were observed in the EBSI and EBS groups. Significant differences were observed among the experimental groups regarding cholesterol and TG levels in the serum of broiler chickens (*p* < 0.05). While serum cholesterol values of the ELP, EBS, ELPI, and EBSI groups were found to be significantly lower compared to the C group (*p* < 0.05), results similar to those of the control were found in the EEF and EEFI groups. The highest (*p* < 0.001) TG level in serum was detected in the C group, followed by the EEF, EEFI, ELPI, and ELP groups, which were found to be similar to each other. The lowest (*p* < 0.001) TG levels were recorded in the EBSI and EBS groups, and were similar to those in the ELPI and ELP groups. No significant (*p* > 0.05) differences were found regarding glucose and TP values in blood serums among the various groups. The differences between TAS and TOS levels in blood serums among the groups were observed to be statistically significant (*p* < 0.001). The lowest (*p* < 0.001) TAS value was observed in the C group, followed by the EEF, EEFI, EBS, EBSI, ELP, and ELPI groups. The EBS, EBSI, ELP, and ELPI groups were found to be statistically similar. The highest (*p* < 0.001) TOS value in blood serum was recorded in the C group, significantly lower and similar values were observed among the ELP, EBSI, EBS, EEF, and EEFI groups, and the lowest values were observed in the ELPI group. IgA and IgM levels in blood serum differed significantly. The highest (*p* < 0.001) IgA values were observed in the ELPI, ELP, EBSI, and EBS groups, followed by the EEFI and EEF groups. The EBS group showed no significant differences compared to the EEF and EEFI groups, while the lowest (*p* < 0.001) values were recorded in the C group. The lowest (*p* < 0.001) IgM value in blood serum was recorded in the C group; significantly lower values were observed in the EEF group, similar to those of the EEFI, EBS, EBSI, and ELP groups; and the highest (*p* < 0.001) values were observed in the ELPI group.

The effect of the dietary supplementation of encapsulated postbiotics derived from different microorganism species alone or with inulin on GH and IGF-1 mRNA expression is presented in Table 7. In comparison to the C group, the supplementation of encapsulated postbiotics alone or with inulin in broilers was up-regulated (*p* < 0.001) in GH and IGF-1 mRNA expression. The highest (*p* < 0.001) values of IGF-1 and GH mRNA expression were observed in the EBSI and EBS groups, followed by the ELPI, ELP, EEFI, and EEF groups.

## 4. Discussion

One of the primary goals of the broiler industry is to enhance growth performance and economically produce animal products. The findings of this study demonstrated that BW on the 21st and 42nd days increased with the supplementation of encapsulated postbiotics, either alone or in combination with inulin. These results are consistent with those of previous studies [20,21]. Several factors may explain this improvement. Postbiotics possess antibacterial properties, both bacteriostatic and bactericidal, that combat pathogenic bacteria and inhibit toxin production in the gut. For instance, Thanh et al. [36] demonstrated that metabolites produced by *Lactobacillus plantarum* inhibit the growth of various pathogens. This antibacterial effect likely reduces subclinical infections, mimicking the effects of antibiotics and, thereby, enhancing nutrient absorption and growth performance. Moreover, postbiotics have been shown to modulate the gut microbiota by promoting the growth of beneficial bacteria and suppressing the colonization of harmful pathogens. This microbiota modulation may improve gut health, increase digestive enzyme activity, and enhance FCR. Furthermore, microbiota modulation, postbiotics contain metabolites such as SCFAs, microbial cell fragments, extracellular polysaccharides, cell lysates, teichoic acids, vitamins, and other bioactive compounds. These metabolites may enhance nutrient absorption by stimulating the development of the intestinal mucosa, particularly the jejunal villi, leading to increased surface area for nutrient uptake. Differences in outcomes among postbiotic-supplemented groups in the present study can likely be attributed to variations in the probiotic strains used. The superior growth performance observed in groups supplemented with *Bacillus subtilis*-derived postbiotics may result from the high production of enzymes during fermentation, which enhances the breakdown of dietary components and improves nutrient bioavailability. Previous studies [20,21] have also reported the growth-promoting effects of *Bacillus subtilis*-derived postbiotics in broilers. Inulin supplementation further enhances growth performance by improving feed digestion and absorption through small intestine histomorphometry modification and by stimulating SCFA production through fermentation in the large intestine [37,38]. SCFAs serve as an energy source for intestinal epithelial cells and modulate gut pH, creating a favorable environment for beneficial bacteria. In this study, improvements in BW on day 21 and BWG during the 1–21-day period were observed in the groups supplemented with inulin and encapsulated postbiotics compared to those supplemented with encapsulated postbiotics alone. This suggests that inulin may synergistically enhance the beneficial effects of postbiotics by improving gut health and nutrient absorption capacity during the starter phase (1–21 days). However, at day 42 in BW and during the 22–42- and 1–42-day periods in BWG, the addition of inulin to encapsulated postbiotic-supplemented groups did not result in statistically significant differences. This may be explained by the fact that the microbiota of broilers is underdeveloped immediately after hatching, and as the microbiota matures over time, the long-term effect of supplementation may diminish. But Kareem et al. [24] reported that supplementing broiler diets with postbiotics (*Lactobacillus plantarum* RG14 and RI11) in combination with inulin (0.8% and 1.0%) resulted in higher BW and BWG compared to diets supplemented with postbiotics alone.

The body weight gain (BWG) of broilers is influenced by various factors, with one of the most direct and fundamental ones being their ability to efficiently convert nutrients from feed into body weight. This efficiency, often referred to as feed efficiency, plays a critical role in determining how effectively broilers transform feed into body mass. In the present study, the supplementation of encapsulated postbiotics, either alone or in combination with inulin, improved feed efficiency throughout all periods of evaluation (days 1–21, 22–42, and 1–42). These findings align with those of Abd El-Ghany et al. [3], who reported that supplementing broiler feed with postbiotics (*Lactobacilli*; Culbac^®^), either alone or in combination with water, significantly enhanced BW and BWG in broilers challenged with *E. coli* (1 × 10⁸ CFU/mL). Similarly, Li et al. [21] observed that postbiotics derived from *Bacillus subtilis* ACCC 11025 at varying levels (0.015%, 0.030%, or 0.045%) significantly improved BW and BWG in broilers. These results support the conclusion that dietary supplementation with postbiotics enhances BWG and feed efficiency, contributing to improved growth performance in broilers.

In the current study, FI was unaffected by the supplementation of postbiotics during days 1–21 but increased during days 22–42 and over the entire experimental period (days 1–42) compared to the C group. The increases in FI and BWG can be explained by improved nutrient absorption, increased secretion of digestive enzymes, and increased villus height, which is associated with the anti-inflammatory effects of postbiotics in chickens. The enhanced gut health and nutrient absorption capacity resulting from postbiotic supplementation might have contributed to better FI and growth performance during the study’s later phases (22–42 and 1–42 days). However, no differences were observed in FI values between the encapsulated groups with or without inulin supplementation during the 22–42- and 1–42-day periods. Kareem et al. [24] reported that the use of postbiotics alone or in combination with inulin did not affect FI (days 1–42). Soren et al. [39] reported that the addition of postbiotics derived from *Saccharomyces cerevisiae* (1.25 g/kg) increased FI during days 1–14, with no significant changes observed over the entire period (days 1–42). This discrepancy could be attributed to differences in the type or strain of probiotics used for postbiotic production, which may lead to variations in their mechanism of action and functional properties. Encapsulated postbiotics, either alone or in combination with inulin, improved FCR during days 1–21. however, no significant impact was observed during days 22–42 or over the entire period (days 1–42). The existing literature contains contradictory findings regarding the impact of postbiotics on FCR in broilers. While some studies have reported improvements [18,21,24], others have found no significant effects [22,40]. These discrepancies in the literature could stem from variations in the types of postbiotics used (derived from different species or strains), differences in the techniques employed during postbiotic production, or differences in the administered supplementation levels.

Other major aims within the broiler industry include increasing the proportion of marketable products relative to the whole carcass weight and improving the yield of edible parts. To this end, the use of feed additives to enhance metabolic rate has become a common practice, as it also improves internal organ development and carcass yield [41]. In the present study, the addition of encapsulated postbiotics significantly improved the yields of carcass, breast, thigh, and abdominal fat. Similarly, Mohammed and Kareem [42] reported that supplementing broiler diets with postbiotics enhanced carcass and breast yields compared to a positive control, although abdominal fat yield remained unaffected. Additionally, Fang et al. [20] observed that supplementing broiler diets with 0.015% postbiotics derived from *Bacillus subtilis* ACCC 11025 improved carcass yields. In contrast, Humam et al. [18] reported no effects on carcass, breast, thigh, or abdominal fat yields in broilers exposed to heat stress. Similarly, Soren et al. [39] found no significant impact of postbiotics on carcass weight, breast weight, or abdominal fat yield in broilers. However, the mechanisms underlying the effects of postbiotics on these carcass traits remain unclear and warrant further investigation. It has been determined that there is no significant difference in carcass characteristics between the use of encapsulated postbiotics alone and their combined use with inulin, indicating that the synergistic effect between them is not reflected in carcass quality. Indeed, this finding is consistent with the results of Kareem et al. [43].

In the present study, the inclusion of encapsulated postbiotics, either alone or in combination with inulin, did not influence the relative weights of the heart, liver, spleen, or pancreas. These results are in agreement with previously conducted studies [18,39,42]. The bursa of Fabricius is a vital immune organ in broiler chickens, and an increase in its relative weight may be indicative of enhanced immune function [21]. In this study, dietary supplementation with encapsulated postbiotics, either alone or in combination with inulin, significantly increased the relative weight of the bursa of Fabricius. The increase in the relative weight of the bursa of Fabricius could be explained by the immunomodulatory effects of postbiotics, which may enhance the development and activity of lymphoid tissues. Postbiotics are known to contain bioactive metabolites such as SCFAs, peptides, and cell wall components, which can stimulate the immune response by promoting the proliferation and differentiation of immune cells. The improved immune function may also result from the modulation of gut microbiota, leading to increased production of beneficial metabolites and a reduction in pathogenic bacterial load, thereby supporting gut-associated lymphoid tissue development and overall immune competence. This observation is consistent with findings by Abd El-Ghany et al. [3], who reported that the inclusion of postbiotics in feed or feed + water added increased the relative weight of the bursa of Fabricius in broilers challenged with *E. coli* Similarly, Mohammed and Kareem [42] noted an increase in the relative weight of the bursa of Fabricius in broilers supplemented with postbiotics. In contrast, studies by Li et al. [21] and Soren et al. [39] found that postbiotics did not impact the weight or relative weight of the bursa of Fabricius. These discrepancies may be attributed to variations in the postbiotic strains used or differences in the dosages of the supplements administered, which could lead to varying levels of metabolite compounds and immunomodulatory effects. These discrepancies may be attributed to variations in the postbiotic strains used or differences in the dosages of the supplements administered. However, the absence of differences between the groups supplemented with encapsulated postbiotics with inulin and those supplemented with encapsulated postbiotics without inulin suggests that inulin did not provide any additional immunomodulatory benefits in this context. This indicates that the observed immune-enhancing effects were primarily driven by the postbiotics themselves rather than a synergistic effect of inulin supplementation.

The physiological response of animals to internal and external conditions, such as nutrition, is often reflected in their hematological profile. In poultry, serum biochemical analysis is a valuable tool for evaluating livers, hearts, and kidneys, as well as the requirements and quality of amino acids and proteins [44]. Blood parameters serve as informative indicators of an animal’s physiological, pathological, and nutritional status. The liver, one of the largest and most essential organs in living organisms, plays a pivotal role in detoxification, metabolism, and the elimination of endogenous and exogenous substances. Diagnostic liver enzyme markers, including ALP, AST, and ALT, are used to assess hepatotoxicity. Elevated AST and ALT levels may indicate pathological conditions, toxicity, or liver damage and dysfunction. In this study, the concentrations of liver enzymes (AST, ALP, GGT, ALT, and LDH) in the serum of broilers supplemented with feed additives were lower than those in the C group. Conversely, Jansseune et al. [40] reported that the addition of postbiotics to two mixed feeds prepared from different raw materials did not influence plasma AST levels. Cholesterol and TG are key indicators of lipid metabolism in serums. In this study, dietary supplementation with encapsulated postbiotics reduced serum cholesterol and TG levels, consistent with the findings of Doski and Kareem [23]. Soren et al. [39] also demonstrated that postbiotic supplementation reduced serum Chol levels in broilers but had no effect on TG levels. Soren et al. [39] and Jansseune et al. [40] both reported that they found no significant changes regarding postbiotic supplementation in the context of serum TP and Glu levels, which aligns with the results of the present study. Furthermore, TAS values in the serum of groups supplemented with encapsulated postbiotics were higher, while their TOS values were lower than those in the C group. These results suggest that postbiotics enhance antioxidant capacity in broilers. He et al. [45] also found that the inclusion of probiotics in broiler diets increased serum TAS levels. One of the objectives of this study was to evaluate the effects of adding postbiotics to broilers’ diets on their immune responses. Immunoglobulins play a critical role in immune system regulation and mucosal defense [18]. These molecules include various subclasses, such as IgM and IgA. IgM primarily functions to regulate the immune response, facilitate the production of the main effector class of immunoglobulins (IgG), and initiate the initial immune response to foreign antigens. In contrast, IgA plays an essential role for mucosal protection by preventing the entry, binding, and colonization of toxins and pathogens. IgA interacts with specific receptors and immune mediators, influencing several protective mechanisms. In the present study, the inclusion of postbiotics in broilers’ diets significantly increased serum IgA and IgM levels. However, Humam et al. [18] observed that postbiotics derived from RI11 in the finisher feed of broilers under heat stress increased plasma IgM levels but did not affect IgA levels.

Hormones play a critical role in determining the growth performance of livestock, with GH and IGF-1 being the primary hormones necessary for maintaining normal growth patterns [46]. Growth hormone contributes to various physiological processes, including growth, aging, reproduction, egg production, and basal metabolic rate. IGF-1, a peptide hormone structurally similar to insulin, is involved in several metabolic and anabolic reactions [46]. The hormonal regulation of growth is complex, involving interactions among various hormones and including the somatotropic axis (GH, growth hormone receptor [GHR], and IGF-1). While GH can directly influence growth, its primary effects are typically mediated by IGF-1 activity. The importance of GH as a regulator of growth and body composition in poultry has been well documented [22]. In poultry, various factors, dietary manipulations in particular, can influence the expression of hormones such as IGF-1 and GH [22]. In the present study, the addition of encapsulated postbiotics, either alone or in combination with inulin, significantly up-regulated GH and IGF-1 expression, in agreement with the findings of Kareem et al. [24] Danladi et al. [22] also reported that the inclusion of postbiotics (0.2%) derived from different *Lactobacillus plantarum* strains (RG11, RG14, RG11, RI11, RS5, and TL1) in broiler diets significantly enhanced the expression of GHR and IGF-1. Additionally, Humam et al. [18] indicated that the supplementation of postbiotics in broiler diets under heat stress up-regulated IGF-1 and GH mRNA expression. Moreover, several studies have demonstrated that postbiotic supplementation can improve gut microbiota [18,20,39] and increase the production of SCFAs [5,24]. In a study on mice, Yan et al. [47] reported that SCFA supplementation increased IGF-1 expression in bone marrow, liver, and adipose tissues. It has been determined that there is no significant difference in GH and IGF-1 levels between the use of encapsulated postbiotics alone and their combined use with inulin, indicating that the synergistic effect between them is not reflected in growth performance. Indeed, our BW data on day 42 and BWG data between days 1 and 42 confirm this finding.

## 5. Conclusions

In conclusion, it was determined that the addition of encapsulated postbiotics (0.30%) derived from Lactobacillus plantarum, Bacillus subtilis, and Enterococcus faecium, either alone or combined with inulin (1.0%), improved growth performance, carcass quality, some blood parameters, GH and IGF-1 mRNA expression, and immune parameters in male broiler chickens. Treatments with postbiotics derived from Bacillus subtilis in encapsulated form alone or with the addition of inulin displayed the best results, especially in terms of growth performance and carcass quality. However, the lack of significant differences between the groups with or without inulin suggests that inulin did not provide additional benefits. The study highlights the potential of postbiotics as alternatives to antibiotic growth promoters, but the cost-effectiveness of encapsulation at a commercial scale requires further evaluation. Early dietary intervention with postbiotics had a greater impact on body weight, with a ~13% increase in the starter period compared to ~12% in the finisher period. It is recommended that future studies investigate the use of postbiotics produced from different probiotic microorganisms and their different combinations and levels as feed additives. In addition, further studies can be carried out to increase the usefulness by developing different coating techniques.

## Figures and Tables

**Table 1:** Compositions and nutrient contents of starter and finisher diets.

Ingredients (g/kg)	Starter (1–21 Day)	Finisher (22–42 Day)
Corn	526.13	638.30
Soybean meal 46CP	254.14	235.01
Sunflower meal 35 CP	30.00	-
Corn gluten meal 62CP	28.00	34.55
Canola meal 34.5CP	21.00	-
Wheat broken	60.00	18.03
DDGS	20.00	-
Meat and bone meal	20.00	36.51
Acid oil	10.00	25.00
Salt	1.80	1.77
DCP	9.28	-
Calcium carbonate	6.22	-
Sodium bicarbonate	1.77	1.15
Methionine	2.80	2.48
Lysine	3.61	3.11
Vitamins and minerals premix ^1^	2.50	2.50
Cholin chloride, mash 70%	1.24	0.99
Threonine 98	1.01	0.60
Anticoccidial	0.50	-
**Calculated composition**		
Dry matter %	88.16	88.02
Ash %	5.53	4.25
Crude protein %	22.04	20.00
Crude fat %	3.63	5.22
Crude fiber %	3.50	2.57
Metabolic energy Kcal/kg diet	2882	3120
Lysine %	1.31	1.17
Methionine %	0.63	0.56
Methionine + cystine %	0.99	0.88
Tryptophan %	0.24	0.20
Calcium %	0.90	0.63
Available Phosphor %	0.52	0.41

Vitamin–mineral premix ^1^ provided per kg of the complete diet: vitamin A, 5,000,000 IU; vitamin D_3_, 1,200,000 IU; vitamin E, 140,000 mg; vitamin K_3_, 1800 mg; vitamin B_1_, 1800 mg; vitamin B_2_, 3000 mg; vitamin B_6_, 2080 mg; vitamin B_12_, 12 mg; D-biotin, 60 mg; folic acid, 1000 mg; niacin, 24,000 mg; calcium-D pantothenate, 5600 mg; manganese (manganese oxide), 32,000 mg; iron (iron sulfate monohydrate), 20,000 mg; copper (copper sulfate pentahydrate), 2000 mg; iodine (calcium iodate anhydride), 400 mg; cobalt (cobalt carbonate monohydrate), 60 mg; zinc (zinc oxide), 30,000 mg; selenium (sodium selenite), 40 mg. DDGS; dried distillers grains with solubles.

**Table 2:** The primer sequences of GH, IGF-1, and ACTB genes used for RT-qPCR.

Gene	Primer Name	Primer Sequence (5′-3′)	Accession No	Product Length (bp)
IGF-1	F	CAGTTCGTATGTGGAGACAGAG	>NM_001004384.3	103
	B	AGCAGCACTCATCCACTATTC	>NM_001004384.3	103
GH	F	GGACTGATGGAAACCTCACTAC	>NM_001397092.1	84
	B	CCGGACATTCTTTCCAGTCTT	>NM_001397092.1	84
ACTB	F	TGGGCCAGAAAGACAGCTAC	>NM_205518.1	82
	B	CCGTGTTCAATGGGGTACTT	>NM_205518.1	82

GH, growth hormone; IGF-1, insulin-like growth factor; ACTB, actin beta.

**Table 3:** Effect of postbiotics derived from different probiotic microorganisms in encapsulated form, alone or with inulin, on growth performance in broiler chickens.

Items	^1^ Treatment							SEM ^2^	*p*
	C	ELP	EBS	EEF	ELPI	EBSI	EEFI		
BW, g									
initial	40.29	41.34	40.64	40.84	40.93	41.14	40.80	0.13	0.429
days 21	647.67 ^f^	670.14 ^c^	696.34 ^a^	658.45 ^e^	676.54 ^b^	698.81 ^a^	667.22 ^d^	2.68	<0.001
days 42	2203.29 ^c^	2293.19 ^b^	2348.11 ^a^	2268.38 ^b^	2300.81 ^b^	2352.45 ^a^	2279.18 ^b^	8.98	<0.001
BWG, g									
days 1–21	607.37 ^f^	628.80 ^c^	655.68 ^a^	617.61 ^e^	635.61 ^b^	657.67 ^a^	626.42 ^d^	2.65	<0.001
days 22–42	1555.62 ^b^	1623.05 ^a^	1653.78 ^a^	1609.92 ^a^	1624.26 ^a^	1655.64 ^a^	1611.95 ^a^	6.90	<0.001
days 1–42	2162.99 ^c^	2251.85 ^b^	2307.46 ^a^	2227.54 ^b^	2259.87 ^b^	2312.31 ^a^	2238.38 ^b^	8.90	<0.001
FI, g									
days 1–21	898.78	904.78	917.32	902.44	905.44	920.09	904.29	2.25	0.083
days 22–42	2572.82 ^b^	2664.15 ^a^	2676.21 ^a^	2637.59 ^a^	2670.77 ^a^	2676.21 ^a^	2650.54 ^a^	8.95	0.013
days 1–42	3471.60 ^b^	3568.45 ^a^	3593.53 ^a^	3540.05 ^a^	3576.23 ^a^	3596.30 ^a^	3555.33 ^a^	9.85	0.005
FCR									
days 1–21	1.48 ^a^	1.44 ^b^	1.40 ^c^	1.46 ^b^	1.42 ^bc^	1.40 ^c^	1.44 ^b^	0.00	<0.001
days 22–42	1.65	1.64	1.62	1.64	1.64	1.62	1.64	0.00	0.801
days 1–42	1.60	1.58	1.56	1.59	1.58	1.56	1.59	0.00	0.075

BW, body weight; BWG, body weight gain; FI, feed intake; FCR, feed conversion ratio. ^1^ C, basal diet; ELP, basal diet + (0.30%) encapsulated postbiotic *Lactobacillus plantarum*; EBS, basal diet + (0.30%) encapsulated postbiotic *Bacillus subtilis*; EEF, basal diet + (0.30%) encapsulated postbiotic *Enterecoccus faecium*; ELPI, basal diet + (0.30%) encapsulated postbiotic *Lactobacillus plantarum* + (1.0%) inulin; EBSI, basal diet + (0.30%) encapsulated postbiotic *Bacillus subtilis* + (1.0%) inulin; EEFI, basal diet + (0.30%) encapsulated postbiotic *Enterecoccus faecium* + (1.0%) inulin. ^a–f^: Means in the same row with different superscript indicates a significant difference (*p* < 0.05), while no superscript indicates no significant difference among groups (*p* > 0.05). ^2^ SEM, standard error of the mean.

**Table 4:** Effect of postbiotics derived from different microorganisms in encapsulated form alone or with inulin on carcass weight and yield in broiler chickens.

Items	^1^ Treatment							SEM ^2^	*p*
	C	ELP	EBS	EEF	ELPI	EBSI	EEFI		
Carcass weight, g	1459.83 ^d^	1554.42 ^b^	1620.58 ^a^	1513.33 ^c^	1559.83 ^b^	1621.67 ^a^	1520.83 ^c^	6.03	<0.001
Carcass yield, %	65.70 ^d^	67.74 ^b^	68.83 ^a^	66.56 ^c^	67.76 ^b^	69.00 ^a^	66.71 ^c^	0.13	<0.001
Breast, g	560.50 ^d^	614.17 ^b^	649.50 ^a^	596.83 ^c^	620.83 ^b^	654.67 ^a^	600.17 ^c^	3.45	<0.001
Breast yield, %	25.23 ^c^	26.76 ^b^	27.69 ^a^	26.25 ^b^	26.97 ^b^	27.83 ^a^	26.33 ^b^	0.06	<0.001
Thigh, g	425.17 ^d^	468.50 ^b^	490.67 ^a^	454.92 ^c^	470.67 ^b^	491.17 ^a^	457.84 ^c^	2.36	<0.001
Thigh yield, %	19.14 ^c^	20.67 ^ab^	21.62 ^a^	20.04 ^b^	20.80 ^ab^	21.76 ^a^	20.33 ^b^	0.02	<0.001
Abdominal fat, g	10.50	10.42	10.37	10.45	10.42	10.37	10.43	0.01	0.267
Abdominal fat yield, %	0.49 ^a^	0.46 ^c^	0.44 ^d^	0.47 ^b^	0.45 ^c^	0.44 ^d^	0.47 ^b^	0.01	<0.001

^1^ C, basal diet; ELP, basal diet + (0.30%) encapsulated postbiotic *Lactobacillus plantarum*; EBS, basal diet + (0.30%) encapsulated postbiotic *Bacillus subtilis*; EEF, basal diet + (0.30%) encapsulated postbiotic *Enterecoccus faecium*; ELPI, basal diet + (0.30%) encapsulated postbiotic *Lactobacillus plantarum* + (1.0%) inulin; EBSI, basal diet + (0.30%) encapsulated postbiotic *Bacillus subtilis* + (1.0%) inulin; EEFI, basal diet + (0.30%) encapsulated postbiotic *Enterecoccus faecium* + (1.0%) inulin. ^a–d^: Means in the same row with different superscript indicates a significant difference (*p* < 0.05), while no superscript indicates no significant difference among groups (*p* > 0.05). ^2^ SEM, standard error of the mean (n:12).

**Table 5:** Effect of postbiotics derived from different microorganisms, in encapsulated form alone or with inulin, on the relative weights of internal organs in broiler chickens.

Items	^1^ Treatment							SEM ^2^	*p*
	C	ELP	EBS	EEF	ELPI	EBSI	EEFI		
Heart, %	0.49	0.50	0.50	0.50	0.50	0.50	0.50	0.00	0.342
Liver, %	2.02	2.06	2.08	2.05	2.06	2.08	2.06	0.00	0.228
Spleen, %	0.14	0.15	0.15	0.15	0.15	0.15	0.15	0.00	0.386
Bursa of Fabricius, %	0.13 ^b^	0.15 ^a^	0.15 ^a^	0.15 ^a^	0.15 ^a^	0.15 ^a^	0.15 ^a^	0.00	0.002
Pancreas, %	0.24	0.25	0.25	0.25	0.25	0.25	0.25	0.00	0.272

^1^ C, basal diet; ELP, basal diet + (0.30%) encapsulated postbiotic *Lactobacillus plantarum*; EBS, basal diet + (0.30%) encapsulated postbiotic *Bacillus subtilis*; EEF, basal diet + (0.30%) encapsulated postbiotic *Enterecoccus faecium*; ELPI, basal diet + (0.30%) encapsulated postbiotic *Lactobacillus plantarum* + (1.0%) inulin; EBSI, basal diet + (0.30%) encapsulated postbiotic *Bacillus subtilis* + (1.0%) inulin; EEFI, basal diet + (0.30%) encapsulated postbiotic *Enterecoccus faecium* + (1.0%) inulin. ^a,b^ Means in the same row with different superscript indicates a significant difference (*p* < 0.05), while no superscript indicates no significant difference among groups (*p* > 0.05). ^2^ SEM, standard error of the mean (n:12).

**Table 6:** Effects of postbiotics derived from different microorganisms, in encapsulated form or inulin, on some blood parameters in broiler chickens.

Items	^1^ Treatment							SEM ^2^	*p*
	C	ELP	EBS	EEF	ELPI	EBSI	EEFI		
AST, U/L	228.90 ^a^	214.73 ^bc^	211.33 ^c^	219.10 ^b^	213.29 ^bc^	210.03 ^c^	218.31 ^b^	1.17	<0.001
ALT, U/L	22.88 ^a^	21.43 ^b^	21.30 ^b^	21.67 ^b^	21.39 ^b^	21.30 ^b^	21.51 ^b^	0.12	0.001
ALP, U/L	3553.67 ^a^	2942.00 ^b^	2880.00 ^b^	3007.50 ^b^	2941.67 ^b^	2878.33 ^b^	3001.33 ^b^	57.02	<0.001
GGT, U/L	16.67 ^a^	13.53 ^b^	13.16 ^bc^	14.50 ^b^	13.33 ^b^	12.67 ^c^	14.00 ^b^	0.24	<0.001
LDH, U/L	864.50 ^a^	756.83 ^c^	749.83 ^d^	767.83 ^b^	753.17 ^c^	749.50 ^d^	765.17 ^bc^	6.16	<0.001
Chol, mg/dL	119.35 ^a^	115.50 ^bc^	112.17 ^c^	116.33 ^ab^	115.33 ^bc^	112.16 ^c^	116.33 ^ab^	0.57	0.003
TG, mg/dL	39.50 ^a^	35.67 ^bc^	34.67 ^c^	35.83 ^b^	35.17 ^bc^	34.33 ^c^	35.80 ^b^	6.90	<0.001
Glu, mg/dL	171.33	165.67	164.33	166.00	164.67	164.00	165.50	0.78	0.182
TP, g/dL	3.71	3.67	3.64	3.67	3.64	3.62	3.68	0.01	0.076
TAS, mmol/L	2.00 ^c^	2.33 ^a^	2.21 ^ab^	2.16 ^b^	2.34 ^a^	2.25 ^ab^	2.17 ^b^	0.02	<0.001
TOS, µmol/L	8.11 ^a^	6.52 ^bc^	6.89 ^b^	6.94 ^b^	6.41 ^c^	6.62 ^bc^	6.92 ^b^	0.09	<0.001
IgA, mg/dL	10.38 ^c^	11.63 ^a^	11.46 ^ab^	11.32 ^b^	11.66 ^a^	11.60 ^a^	11.38 ^b^	0.07	<0.001
IgM, mg/dL	6.55 ^c^	8.15 ^ab^	8.04 ^ab^	7.87 ^b^	8.24 ^a^	8.09 ^ab^	7.96 ^ab^	0.09	<0.001

AST, aspartate aminotransferase; ALT, alanine aminotransferase; ALP, alkaline phosphatase; GGT, gamma-glutamyl transferase; LDH, lactate dehydrogenase; Chol, cholesterol; TG, triglyceride; Glu, glucose; TP, total protein; TAS, total antioxidant status; TOS, total oxidant status; IgA, immunoglobulin A; IgM, immunoglobulin M. ^1^ C, basal diet; ELP, basal diet + (0.30%) encapsulated postbiotic *Lactobacillus plantarum*; EBS, basal diet + (0.30%) encapsulated postbiotic *Bacillus subtilis*; EEF, basal diet + (0.30%) encapsulated postbiotic *Enterecoccus faecium*; ELPI, basal diet + (0.30%) encapsulated postbiotic *Lactobacillus plantarum* + (1.0%) inulin; EBSI, basal diet + (0.30%) encapsulated postbiotic *Bacillus subtilis* + (1.0%) inulin; EEFI, basal diet + (0.30%) encapsulated postbiotic *Enterecoccus faecium* + (1.0%) inulin. ^a–d^ Means in the same row with different superscript indicates a significant difference (*p* < 0.05), while no superscript indicates no significant difference among groups (*p* > 0.05). ^2^ SEM, standard error of the mean (n:12).

**Table 7:** Effect of postbiotics derived from different microorganisms, in encapsulated form or with inulin, on GH and IGF-1 mRNA expression values in broiler chickens.

Items	^1^ Treatment							SEM ^2^	*p*
	C	ELP	EBS	EEF	ELPI	EBSI	EEFI		
GH	1.00 ^c^	3.65 ^b^	3.82 ^a^	3.62 ^b^	3.67 ^b^	3.83 ^a^	3.63 ^b^	0.04	<0.001
IGF-1	1.00 ^c^	5.67 ^b^	6.31 ^a^	5.59 ^b^	5.69 ^b^	6.31 ^a^	5.60 ^b^	0.27	<0.001

^1^ GH, growth hormone; IGF-1, insulin-like growth factor-1. SEM, standard error of the mean. ^2^ SEM, standard error of the mean. C, basal diet; ELP, basal diet + (0.30%) encapsulated postbiotic *Lactobacillus plantarum*; EBS, basal diet + (0.30%) encapsulated postbiotic *Bacillus subtilis*; EEF, basal diet + (0.30%) encapsulated postbiotic *Enterecoccus faecium*; ELPI, basal diet + (0.30%) encapsulated postbiotic *Lactobacillus plantarum* + (1.0%) inulin; EBSI, basal diet + (0.30%) encapsulated postbiotic *Bacillus subtilis* + (1.0%) inulin; EEFI, basal diet + (0.30%) encapsulated postbiotic *Enterecoccus faecium* + (1.0%) inulin. ^a–c^: Means in the same row with different superscript indicates a significant difference (*p* < 0.05), while no superscript indicates no significant difference among groups (*p* > 0.05).

## Data Availability

All data sets collected and analyzed during the current study are available from the corresponding author on fair request.
